# Optimal Strategies for the Surgical and Long‐Term Management of Malignant Struma Ovarii

**DOI:** 10.1155/crie/4120842

**Published:** 2026-02-23

**Authors:** Anna Hayden, Nathaniel Grabill, Mena Louis, Ezra Ellis, Nikita Machado

**Affiliations:** ^1^ Graduate Medical Education Department, Northeast Georgia Health System Inc, Gainesville, Georgia, USA; ^2^ Pathology Department, Northeast Georgia Health System Inc, Gainesville, Georgia, USA; ^3^ FACS, Endocrine Surgery Department, Northeast Georgia Health System Inc, Gainesville, Georgia, USA

**Keywords:** malignant transformation, oophorectomy, ovarian cyst, ovarian tumor, papillary thyroid carcinoma, salpingectomy, struma ovarii, thyroglobulin, thyroidectomy

## Abstract

Struma ovarii is a rare ovarian tumor characterized by the presence of thyroid tissue, which can occasionally undergo malignant transformation. Management varies due to its rarity and the potential for malignancy. A 25‐year‐old female with a history of polycystic ovary syndrome (PCOS) presented with a 7 cm left ovarian cyst. Laparoscopic cystectomy revealed struma ovarii with areas of papillary thyroid carcinoma. Preoperative evaluations, including thyroid ultrasound and PET scan, showed no evidence of primary thyroid cancer or metastatic disease. Given the malignant potential, a total thyroidectomy was recommended to facilitate monitoring of thyroglobulin levels for early detection of recurrence. The patient was also scheduled for left salpingectomy and oophorectomy to reduce reoccurrence and perform a complete oncologic resection which was completed approximately 1 month following thyroidectomy and found to be negative for carcinoma. Struma ovarii requires careful management due to its potential for malignancy. Proactive surgical intervention and regular monitoring are critical to managing recurrence and ensuring patient safety. Thyroglobulin levels serve as an effective biomarker for early detection of any residual disease, guiding follow‐up care. This patient’s initial level of thyroglobulin was 15.2 ng/mL. This was obtained post initial cystectomy but prior to thyroidectomy. Thyroglobulin monitoring and follow‐up are essential for early detection and management of recurrence. This is particularly true for well‐differentiated thyroid cancers, which this is not. However, it can still be an important indicator for the presence of thyroid tissue in other areas which could point to a recurrent malignancy. This case emphasizes the need for a tailored approach in managing struma ovarii, considering the variability in malignant transformation risk.

## 1. Introduction

Struma ovarii, a rare subtype of ovarian teratoma, is characterized by the presence of thyroid tissue within the ovary [1]. While ovarian teratomas are relatively common, accounting for approximately 10%–20% of all ovarian neoplasms, struma ovarii represents only 2%–5% of these cases [2]. The presence of thyroid tissue in an ectopic location presents unique challenges in diagnosis and management, particularly due to its potential for both benign and malignant transformations [3]. To meet criteria for struma ovarii, thyroid tissue must comprise greater than 50% of the teratoma [4].

The clinical presentation of struma ovarii is varied, often mimicking other gynecological conditions, such as ovarian cysts, endometriosis, adenomyosis, or other conditions that cause pelvic pain [5]. Additionally, many patients remain asymptomatic, and the diagnosis is frequently made incidentally during imaging or surgical procedures for other suspected ovarian masses [4]. However, when symptoms do occur, they can range from abdominal pain and a palpable mass to thyroid‐related symptoms such as hyperthyroidism, making the clinical picture more complex [2]. Patients can present with symptoms similar to any other ovarian tumor such as abnormal vaginal bleeding or abnormal menstrual cycles [6]. However this can be difficult to diagnose in patients with comorbid conditions such as polycystic ovary syndrome (PCOS) [7]. This variability in presentation can delay diagnosis and complicate treatment strategies [7].

Management of struma ovarii depends largely on the presence or absence of malignant transformation [4]. Malignant transformation in struma ovarii varies, with rates ranging from 0.17%–10%, based on a review of the literature [8]. Malignant transformation is defined by cytologic or histologic features consistent with a papillary or follicular thyroid carcinoma. For follicular carcinoma, capsular or vascular invasion is required. Malignancy may also be diagnosed based on metastatic spread. Recurrence may be the first clue to malignancy in some cases of follicular carcinoma [5]. While most cases are benign and can be managed with conservative surgery, the risk of malignancy, although low, necessitates a careful and individualized approach [9]. This includes thorough preoperative evaluation, appropriate surgical intervention, and vigilant postoperative monitoring to ensure any potential malignancy is detected and managed promptly [10, 11].

The age that struma ovarii is diagnosed determines management [10]. In premenopausal females, a cystectomy would be indicated to preserve fertility [12]. In postmenopausal women the rates of malignant transformation are higher, and these should be managed with a high index of suspicion [1, 13]. Premenopausal females with this pathology will need to be followed for many years, as malignant transformation occurs most commonly in the 50–60 s age range [3]. The prognosis of patients with malignant struma ovarii is varied and is particularly not documented well in the literature due to loss of follow‐up, as there can be a long interval between detection and malignant transformation [14, 15]. There is data on multiple different methods for treatment of struma ovarii that has undergone malignant transformation [16]. Treatment modalities depend on many factors, age of diagnosis, comorbid conditions, and presence and amount of metastasis [17].

## 2. Case Presentation

**Figure 1:**
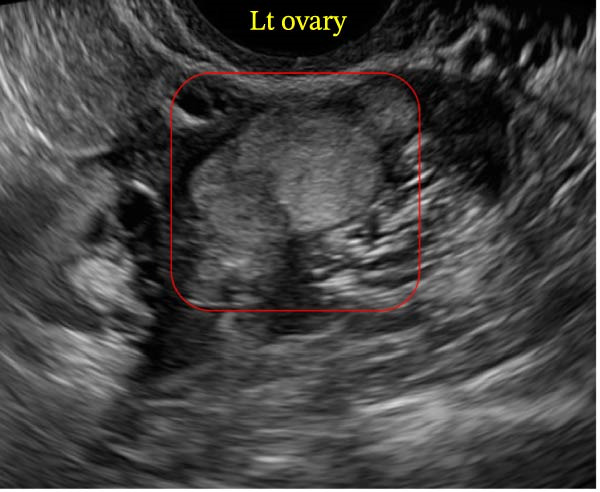
Transvaginal ultrasound showing a hyperechoic lesion in the left ovary. The appearance is consistent with either a hemorrhagic cyst or echogenic thyroid‐like tissue suggestive of struma ovarii. No significant free fluid was observed around the ovaries or in the pouch of Douglas during this examination.

A 25‐year‐old female with a history of PCOS presented with a persistent and enlarging left ovarian cyst. The cyst was initially identified during routine gynecological examination and measured approximately 39.2 mm × 18.3 mm × 26.1 mm by transvaginal ultrasound (Figure 1). The patient reported a history of irregular menstrual cycles and intermittent lower abdominal discomfort, which she attributed to her known diagnosis of PCOS. She denied any symptoms suggestive of thyroid dysfunction, such as weight changes, heat intolerance, or palpitations.

Given the size and persistence of the ovarian cyst, the patient was scheduled for laparoscopic left ovarian cystectomy. Preoperative cystectomy evaluation included routine laboratory tests, which revealed normal levels of CA 125 (7 U/mL), alpha‐fetoprotein (2.9 ng/mL), and lactate dehydrogenase (140 U/L), and complete blood count within normal limits.

**Figure 2:**
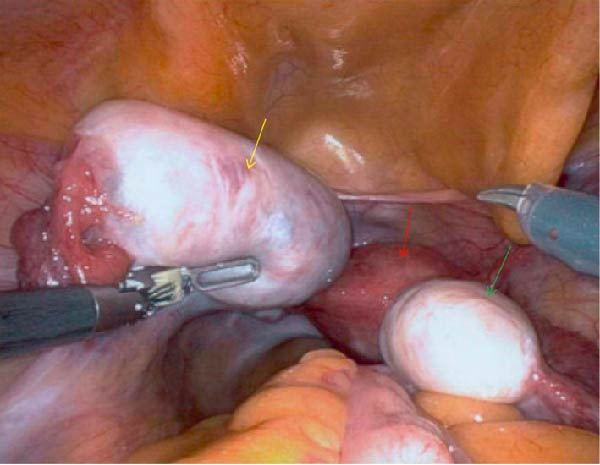
Intraoperative robotic image displaying the ovarian tumor (yellow arrow) in situ. The irregular appearance does not resemble a simple cyst, reflecting the malignant component within this teratoma. Additional anatomical structures noted; uterus (red arrow), right ovary (green arrow).

The patient underwent laparoscopic left ovarian cystectomy. During the procedure, two separate dermoid cysts were identified and removed from the left ovary (Figure 2). The right ovary was noted to be slightly enlarged; however, no cysts were evident upon exploration, and the ovary was closed with sutures to achieve hemostasis. No fluid was noted around the ovaries or in the pouch of Douglas. The surgery proceeded without complications, and the patient recovered well postoperatively.

**Figure 3:**
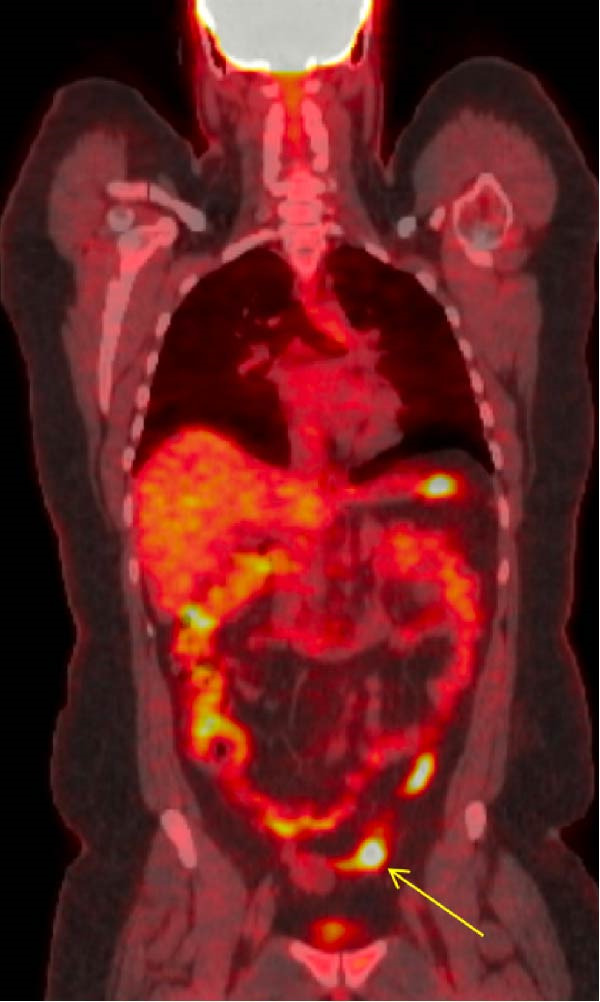
18‐FDG PET CT skull base to mid‐thigh (coronal view) scan showing a minimally avid and mildly prominent left inguinal node (yellow arrow), likely reactive due to its location, with no evidence of metastatic disease. The other areas of reactivity are considered physiologic.

The cystectomy pathology report displayed thyroid tissue after what was thought to only be a routine excision of an ovarian dermoid cyst. This unexpected diagnosis presented the need for further follow‐up imaging. A thyroid ultrasound was performed to rule out any primary thyroid pathology, and it demonstrated a normal‐appearing thyroid gland with no evidence of nodules or masses. An 18 F‐FDG PET/CT scan was performed, demonstrating a minimally avid, mildly prominent left inguinal lymph node, which was interpreted as reactive rather than malignant (Figure 3). No additional lesions suggestive of metastatic disease were identified on the scan.

**Figure 4:**
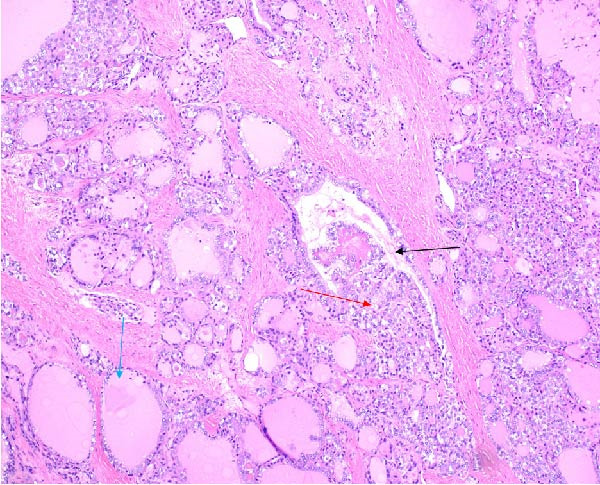
Hematoxylin and eosin (H&E) stain at 10x magnification demonstrating the transition from normal thyroid‐like vesicles to papillary thyroid carcinoma. The blue arrow indicates relatively normal‐appearing follicles, the black arrow indicates abnormal or distorted follicular structures, and the red arrow highlights tumoral papillae with fibrovascular cores lined by neoplastic epithelial cells.

**Figure 5:**
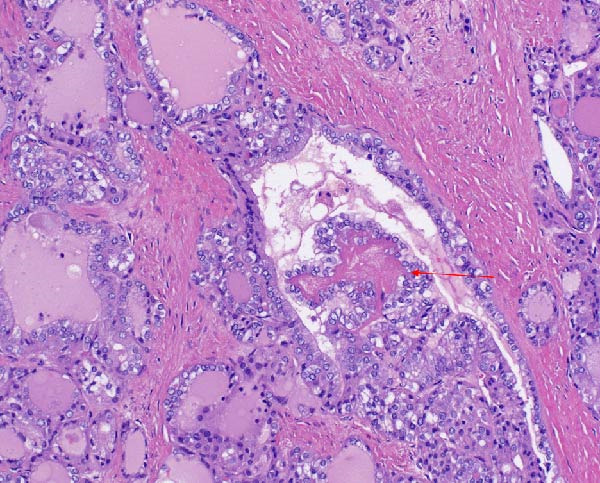
Hematoxylin and eosin (H&E) stain at 20x magnification, providing a closer view of the papillary thyroid carcinoma. Red arrow identifies an area of architectural disruption and nuclear atypia.

The excised ovarian tissue was sent for histopathological examination. Gross examination of the larger cyst revealed features consistent with a mature cystic teratoma. Microscopic analysis demonstrated thyroid tissue with a follicular architecture, interspersed with areas showing features of papillary thyroid carcinoma (Figures 4 and 5). Notably, sections of the tissue displayed solid growth patterns with decreased nuclear features characteristic of papillary thyroid carcinoma and an increased mitotic index, raising concerns for progression to poorly differentiated thyroid carcinoma.

**Figure 6:**
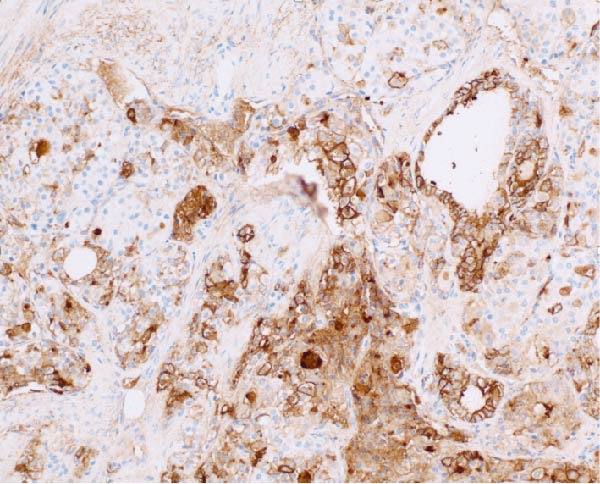
An additional 20x magnification highlights the HBME‐1 immunostaining, which shows focal positivity, which is characteristic utb not required for papillary thyroid carcinoma. Other immunostaining and immunomarkers used to characterize a tumor of thyroid origin include CK19, TTF1 and thyroglobulin as well as others listed in Table 1.

**Figure 7:**
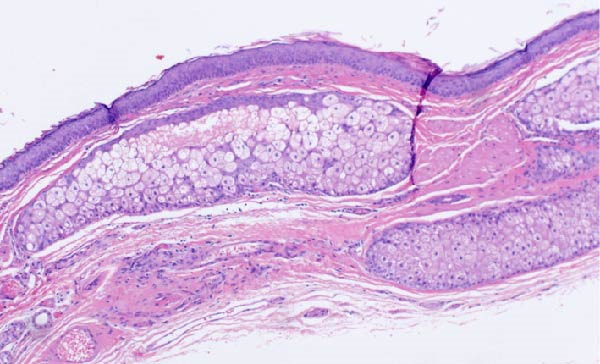
Hematoxylin and eosin (H&E) stain at 10x demonstrating mature teratoma tissue.

Immunohistochemical staining was performed to further characterize the lesion. The BRAF V600E mutation, commonly associated with papillary thyroid carcinoma, was negative. Presence of this mutation has been linked to more aggressive tumor behavior and a poorer prognosis. The NRAS/HRAS Q16R mutation was equivocal, and the Ki‐67 proliferation index was up to 15%, indicating a moderate level of cellular proliferation. This may be associated with a higher risk profile and warrants close monitoring. HBME‐1, a marker often positive in papillary thyroid carcinoma showed focal positive staining (Figure 6). Additional immunostaining with synaptophysin, a neuroendocrine marker, was negative. See Table 1 for various immunostains and typical expression in thyroid and ovarian tissue with their utility in diagnosis. These findings supported the diagnosis of papillary thyroid carcinoma arising within a mature cystic teratoma (Figure 7) with areas worrisome for progression to poorly differentiated thyroid carcinoma. Cell characteristics that move further from the typical appearance of a normal healthy cell point more towards a diagnosis of poor differentiation.

**Table 1:** Key immunostains for identifying ovarian tumors of thyroid origin.

Immunostain	Typical expression in thyroid tissue	Typical expression in ovarian tissue	Utility in struma ovarii/papillary thyroid carcinoma
Thyroglobulin (Tg)	Strongly positive in normal thyroid tissue and well‐differentiated thyroid carcinomas	Usually negative in nonthyroid ovarian tumors	Confirms thyroid origin when present in an ovarian tumor
TTF‐1	Positive in most thyroid carcinomas	Typically, negative in epithelial ovarian tumors (except in rare metastatic lung lesions)	Helps confirm thyroid lineage
HBME‐1	Frequently positive in papillary thyroid carcinoma	Not characteristically expressed in benign ovarian tissue	Suggests papillary thyroid carcinoma when focal or diffuse positivity is observed
Cytokeratin 7 (CK7)	Commonly positive in thyroid carcinoma	Also positive in various ovarian carcinomas (e.g., serous)	May support diagnosis when used in combination with more specific markers
Cytokeratin 20 (CK20)	Usually negative in thyroid carcinoma	Usually negative or variable in ovarian tumors	Not discriminatory alone; must be interpreted alongside other markers
Synaptophysin	Negative in thyroid carcinoma	Positive in neuroendocrine components	Useful for ruling out neuroendocrine differentiation within the teratoma
PAX8	Usually positive in thyroid carcinoma	Also expressed in Müllerian (ovarian) tumors	Can assist in confirming thyroid tissue origin in the context of other markers

Following the unexpected pathology result, the patient was scheduled for a left salpingectomy, oophorectomy and total thyroidectomy to allow surveillance using serum thyroglobulin (Tg) levels. The pre‐thyroidectomy Tg level was 15 ng/mL, dropping to 1.5 ng/mL post‐thyroidectomy, and 1.8 ng/mL at the 6‐week follow‐up. Pre‐thyroidectomy TSH and Free T4 were within normal limits, and thyroglobulin antibodies level was <1.0 IU/mL, remaining so post‐thyroidectomy. Molecular testing was not completed.

## 3. Discussion

This case initially presented as a typical dermoid cyst, a common ovarian mass frequently encountered in clinical practice. Ovarian teratomas are relatively common, accounting for approximately 10%–20% of all ovarian neoplasms, while struma ovarii represents only 2%–5% of these cases. Despite the seemingly benign ultrasound appearance, malignant transformation can occur, altering both prognosis and management [3, 18, 19]. Several publications have described lethal or widely metastatic malignant struma ovarii, underscoring the potential severity in some patients [20–22].

In the present case, histopathological analysis revealed papillary thyroid carcinoma within the ovarian teratoma, exhibiting solid growth patterns and a higher mitotic index in certain areas. These features raise concern for progression toward a poorly differentiated subtype, known to correlate with an elevated risk of local recurrence and distant spread [17, 23]. Poorly differentiated thyroid carcinoma often shows reduced nuclear characteristics typical of papillary thyroid carcinoma (such as nuclear grooves and pseudoinclusions), instead demonstrating more aggressive histologic traits and higher proliferative activity, prompting a more assertive therapeutic plan.

Along with the ovarian cystectomy, the patient was also scheduled for left salpingectomy and oophorectomy to decrease the likelihood of recurrence and achieve a more complete oncologic resection [19, 24]. A total thyroidectomy was undertaken to remove native thyroid tissue and enable precise surveillance using serum thyroglobulin (Tg) levels [25]. In this patient, the preoperative Tg level was 15 ng/mL, dropping to 1.5 ng/mL post‐thyroidectomy, and measured at 1.8 ng/mL at the 6‐week follow‐up. These findings suggest effective removal of thyroid‐origin tissue. Persistent or rising Tg levels in subsequent follow‐up would signal residual or recurrent disease, prompting additional interventions.

Tg serves as a key biomarker in the management of thyroid carcinoma, including malignant struma ovarii. Systematic Tg assessment is not always performed in cases where the thyroid ultrasound has a benign appearance; however, in patients with any suspicion of ectopic thyroid tissue or atypical ovarian masses, Tg measurement can help establish a baseline and monitor treatment response or early recurrence [26, 27]. When facing a teratoma that contains thyroid tissue, especially with malignant features, Tg monitoring becomes integral to long‐term care. Imaging techniques, such as ultrasound or computed tomography, may fail to detect microscopic disease, whereas Tg level fluctuations can indicate a need for further evaluation.

This patient will undergo postoperative radioactive iodine (RAI) therapy due to her young age and the desire for an aggressive approach in the context of potentially poorly differentiated malignant struma ovarii [17]. RAI targets any remaining thyroid‐origin cells by delivering cytotoxic radiation through iodine uptake. However, in cases of dedifferentiation—referred to as the “switch phenomenon”—the malignant cells may lose the ability to concentrate iodine, limiting RAI efficacy [21]. When this occurs, 18F‐FDG PET can identify metabolically active lesions that do not appear on standard iodine‐based imaging. Early detection of such lesions is critical, as these may require alternate therapies.

Although most patients respond to surgical resection and RAI, some may develop metastatic disease or experience recurrence. In those situations, treatment options can include external beam radiation, chemotherapy, or targeted therapies such as tyrosine kinase inhibitors (TKIs). These novel agents (e.g., sorafenib and lenvatinib) have shown benefit in advanced or iodine‐refractory thyroid carcinomas, though evidence in malignant struma ovarii is limited [5, 25, 28]. Close follow‐up is vital, involving regular Tg measurement and imaging to detect disease progression early. A persistent or rising Tg level in the absence of iodine‐avid lesions on scans should prompt further investigation with FDG PET to locate any occult dedifferentiated metastases.

The prognosis in malignant struma ovarii varies widely. Many patients remain disease‐free after complete resection and RAI, whereas others may experience relapse or distant spread [20, 29]. Factors influencing outcomes include the extent of disease at diagnosis, histological features (especially the degree of differentiation), and response to RAI therapy. Thorough surgical removal, vigilant Tg monitoring, and early detection of potential dedifferentiation are crucial for improving long‐term survival. This patient’s course will continue to be monitored via Tg levels and yearly imaging, aiming for timely intervention should recurrence or metastasis emerge.

## 4. Conclusion

Struma ovarii is a rare ovarian tumor with the potential for malignant transformation. Total thyroidectomy enables monitoring of thyroglobulin levels to detect any residual or recurrent thyroid tissue. Given the variability in the risk of malignancy, careful postoperative surveillance is essential. The management plan, including thyroidectomy, salpingectomy, and oophorectomy, aims to minimize future complications and improve outcomes. Close postoperative surveillance is essential due to the long interval between detection and malignant transformation to prevent loss to follow‐up. Malignant transformation most often occurs in the 5^th^ or 6^th^ decade of life, therefore these patients end up being followed for many years. Unfortunately, due to the long length of follow‐up required, many of these patients end up being lost to follow‐up.

## Author Contributions

**Anna Hayden**: data collection, manuscript author, manuscript review, patient consent. **Nathaniel Grabill**: figure generation, manuscript author, manuscript review, project management. **Mena Louis**: manuscript author, manuscript review. **Ezra Ellis**: manuscript author, manuscript review, pathology figure generation. **Nikita Machado**: manuscript author, manuscript review.

## Funding

No funding was provided for support of this article.

## Disclosure

An earlier version of this manuscript was presented at an internal‐only graduate medical education research day at the Northeast Georgia Medical Center as a poster presentation. The link to this presentation is below: https://event.fourwaves.com/nghs2025/abstracts/d956e7e3-bbdc‐48f1‐934e‐0943d3503f2d.

## Consent

All the patients allowed personal data processing and informed consent was obtained from all individual participants included in the study.

## Conflicts of Interest

The authors declare no conflicts of interest.

## Data Availability

The data that support the findings of this study are available from the corresponding author upon reasonable request.
